# Utilising Commercially Fabricated Printed Circuit Boards as an Electrochemical Biosensing Platform

**DOI:** 10.3390/mi12070793

**Published:** 2021-07-03

**Authors:** Uroš Zupančič, Joshua Rainbow, Pedro Estrela, Despina Moschou

**Affiliations:** Centre for Biosensors, Bioelectronics and Biodevices (C3Bio), Department of Electronic & Electrical Engineering, University of Bath, Claverton Down, Bath BA2 7AY, UK; uz206@bath.ac.uk (U.Z.); jr993@bath.ac.uk (J.R.); p.estrela@bath.ac.uk (P.E.)

**Keywords:** printed circuit boards, electrochemical biosensors, Lab-on-PCB, electrode pre-treatment

## Abstract

Printed circuit boards (PCBs) offer a promising platform for the development of electronics-assisted biomedical diagnostic sensors and microsystems. The long-standing industrial basis offers distinctive advantages for cost-effective, reproducible, and easily integrated sample-in-answer-out diagnostic microsystems. Nonetheless, the commercial techniques used in the fabrication of PCBs produce various contaminants potentially degrading severely their stability and repeatability in electrochemical sensing applications. Herein, we analyse for the first time such critical technological considerations, allowing the exploitation of commercial PCB platforms as reliable electrochemical sensing platforms. The presented electrochemical and physical characterisation data reveal clear evidence of both organic and inorganic sensing electrode surface contaminants, which can be removed using various pre-cleaning techniques. We demonstrate that, following such pre-treatment rules, PCB-based electrodes can be reliably fabricated for sensitive electrochemical biosensors. Herein, we demonstrate the applicability of the methodology both for labelled protein (procalcitonin) and label-free nucleic acid (*E. coli*-specific DNA) biomarker quantification, with observed limits of detection (LoD) of 2 pM and 110 pM, respectively. The proposed optimisation of surface pre-treatment is critical in the development of robust and sensitive PCB-based electrochemical sensors for both clinical and environmental diagnostics and monitoring applications.

## 1. Introduction

While the first idea for printed circuit boards originated in the early 1930s, the industry has come a significant way and expanded into almost every sector within the field of electronics, becoming a ubiquitous part of our everyday lives. Printed circuit boards (PCBs) have inarguably opened the door to the huge technological expansion and development of our times. Thus, there has been constant pressure for technological advancement, pushing for improved PCB performance, cost-effectiveness, and miniaturisation, over the last twenty-five years [1]. One application area facilitated by this advancement in high-specification commercial PCB technology is the sensors and diagnostics field, including the development of biosensors for the detection of biomolecules in clinical and environmental applications [2]. While first stipulated in the 1990s as a concept for integrated microchips for biomolecular detection [3], PCBs have recently re-emerged as a promising platform for the development of fully integrated and electronics-enabled Lab-on-Chip (LoC) platforms. To date, a large proportion of the conducted research has been focusing on microfluidic component and biosensor prototyping by complementary metal oxide semiconductor (CMOS) and polymer material platforms [4]. However, the development of LoC devices addressing demanding biomedical applications requires the integration of not only electronic sensor components but also sample preparation microfluidic components, heating, and filtration elements as well as fluid actuators and a user-friendly interface with the capability of data transmission and storage. The inherent characteristic of PCBs to be easily integrated with electronics in cm-scale devices in an unscalable and modular fashion has proven to be a distinct advantage, fuelling its recent re-emergence as Lab-on-PCB technology.

The concept of Lab-on-PCB devices combines high sensor performance (sensitivity and selectivity), cost-effectiveness, and the ability to be easily integrated [5]. Lab-on-Chip devices should meet the REASSURED criteria for point-of-care diagnostics if they are to be used for clinical purposes. This requires the sensor to be capable of real-time analysis, with ease of sample collection, affordability, sensitivity, specificity, user-friendliness, speed, equipment-free, and to be capable of being delivered to the patient within their location [5,6,7]. PCBs can realise real-time analysis through the interfacing of sensing electrodes and miniaturised electronics for electronic data acquisition, transmission, and storage.

Despite these unique advantages, new technological hurdles have emerged while developing commercial Lab-on-PCB platforms, arising from the fact that the industrial PCB fabrication process was not initially designed for use in the context of diagnostics. Thus, there is a major question that needs to be addressed when utilising these devices for highly sensitive and reliable electrochemical sensors. The fabrication process of commercial PCBs is highly industrial and comes with a plethora of contaminants that must be removed before use. These contaminants exist in both organic and inorganic forms and can cause issues including low sensitivity and signal interference. Issues can also arise in low fidelity with certain surface chemistry functionalisation techniques if not properly pre-cleaned before use.

This paper aims to characterise both the electrochemical and physical properties of PCB gold electrodes fabricated using a standard, industrial process (hard gold plating). We take a specific interest in the post-fabrication contamination and techniques for minimising it prior to constructing an electrochemical biosensor, as well as the sensing electrode surface roughness parameter. Finally, the paper proves the validity of these approaches, demonstrating two examples of sensitive PCB-based electrochemical biosensors for the detection of clinically relevant protein and nucleic acid biomarkers.

## 2. Materials and Methods

Hydrogen peroxide, ammonium hydroxide, potassium hydroxide, potassium ferrocyanide and ferricyanide, phosphate-buffered saline tablets, potassium chloride, and copper etchant (CE-100) were purchased from Sigma-Aldrich (Gillingham, UK), while 1 M sulphuric acid was purchased from Thermo Fisher Scientific (Loughborough, UK). The Ag/AgCl (KCl) reference electrode was purchased from BASi (West Lafayette, IN, USA) and the platinum wire used as the counter-electrode was obtained from ALS (Tokyo, Japan). Milli-Q water was obtained using the Millipore Direct-Q 5 UV Water Purification System and deionised (DI) water. Oxygen plasma Zepto System (Diener electronic, Ebhausen, Germany) was used to perform PCB plasma cleaning.

PCBs were designed using Altium Designer 18 software and fabricated by Lyncolec (Dorset, UK). In short, 1.6 mm FR-4 covered with 1 oz copper was patterned and plated with hard gold by electrodeposition of nickel (3–5 µm) and gold (1 µm) and outlined by the solder mask to make PCB electrodes with 1 mm diameter. PCB boards used in the study are shown in Figure S1. Pictures were taken using a Huawei P10 smartphone.

### 2.1. Surface Roughness Characterisation

AFM analysis was performed using Digital Instruments Nanoscope IIIA, and Gwyddion software was used for image analysis and profile extraction. SPR chips (Reichert Technologies, Buffalo, NY, USA) coated with a thin gold layer through vacuum deposition were used as planar electrodes for surface characterisation. For electrochemical characterisation, the chip was first cleaned using piranha solution (9 mL of 99.9% sulphuric acid, mixed with 3 mL of 30% hydrogen peroxide, for 5 min) before washing in MQ water. Electrodes were outlined by double-sided adhesive (300LSE, 3M, Bracknell, UK), where a 2 mm diameter circular hole was cut using a puncher. Then, 25 µL of 50 mM H_2_SO_4_ was deposited on the outlined electrode and contacted with the Ag/AgCl (KCl) reference electrode and platinum wire counter-electrode. Sulphuric acid cycling was performed using an Ag/AgCl (KCl) reference electrode and platinum wire counter-electrode between −0.2 and +1.5 V at 200 mV/s. Roughness factor was calculated by integration of the gold-oxide reduction peak as described previously [8] by calculation of electrochemical surface area (*ESA*):

$$Q=\frac{1}{v_{r}}\int i\cdot V\prime dV\prime$$

where *Q* is the charge, *v_r_* is the scan rate, and the integral ∫i·V′dV′ is the area of the gold oxide reduction peak. *ESA* can then be determined by:

$$ESA=\frac{Q}{390\cdot 10^{-6}}$$

Furthermore, the surface roughness factor (*R_f_*) can be calculated:

$$R_{f}=\frac{ESA}{A}$$

PCBs were cleaned using a LT SC-1 cleaning procedure before AFM analysis, followed by cycling in H_2_SO_4_ as described above to acquire surface roughness factors.

### 2.2. PCB Cleaning and Electrochemical Analytical Techniques

PCB boards represented in Figure S1 were used in the cleaning optimisation study. The cleaning procedures are described in Table 1 and Table S1.

Oxidation and reduction of potassium ferri-/ferrocyanide was evaluated by cyclic voltammetry (CV) in 5 mM ferri-/ferrocyanide couple in PBS with 1 M KCl, scanning between −0.2 and 0.7 V vs. Ag/AgCl (KCl) at 100 mV/s scan rate. The current density was obtained by integration of oxidation peak and dividing the peak height with the geometrical area of the electrode. Charge transfer resistance was obtained by EIS scan in the abovementioned solution by scanning from 100,000 Hz to 1 Hz at 10 mV amplitude at the DC bias of the formal potential observed in a CV (e.g., 0.245 V vs. Ag/AgCl (KCl)). The obtained plot was fitted with Randles equivalent circuit to extract the Rct value. For evaluation of the impurity peaks, CV was performed in PBS from −0.3 to 0.8 V vs. Ag/AgCl (KCl) at 1 V/s scan rate and peaks at approximately 0.35 V vs. Ag/AgCl (KCl) were evaluated.

### 2.3. Electrochemical Sensor for E. coli DNA Detection 

PCB electrodes were cleaned utilising the low-temperature standard clean-1 (LT SC-1) method as detailed above. Following this cleaning, electrodes were passively functionalised using an optimised 1:15 molar ratio of 1 µM thiolated single stranded peptide nucleic acid (ssPNA) and 1 µM 6-Mercapto-1-Hexanol (MCH) in 50% dimethyl sulfoxide (DMSO) diluted in Milli-Q (18.2 MΩ.cm at 25 °C). This was performed at 4 °C for approximately 16 h to form a self-assembled monolayer as described previously [5]. After functionalisation, electrodes were rinsed with Milli-Q and backfilled with 1 mM MCH diluted in 10 mM phosphate buffer (PB) for 50 min at room temperature. Electrodes were then rinsed with Milli-Q and incubated in measurement buffer (10 mM PB containing 4 mM [Fe(CN)_6_]^3/4-^) for 2 h to stabilise the self-assembly monolayer (SAM). The target ssDNA sequence was diluted in 10 mM PB and heated to 95 °C for 5 min prior to incubation. A sample volume of 10 µL was incubated on each electrode for 30 min before measuring signal change upon hybridisation using electrochemical impedance spectroscopy (EIS). The measurements were taken using the on-board three-electrode setup with a gold PCB quasi-reference electrode. Thus, the EIS spectra were scanned between 100,000 and 0.1 Hz with no DC bias and potential amplitude of 0.01 V versus open circuit potential (OCP). The calibration curve data were fit with a non-linear curve fitting. Change from the blank was subtracted before averaging data and fitting with Randle’s equivalent circuit to obtain charge transfer resistance (ΔR_ct_).

### 2.4. Electrochemical Detection of PCT Protein

ELISA was performed using PCT antibody pair (Abcam, Cambridge, UK, part no. ab222276) with Nunc MaxiSorp^TM^ high protein-binding 96-well ELISA plates by diluting the capturing antibody to 2 µg/mL in PBS and coating the plate for 2 h (100 µL/well) while shaking (200 rpm). Blocking was performed using 1% BSA in PBS for 1 h while shaking. PCT serial dilution was performed according to the manufacturer’s protocol and the detection antibody was incubated with the target at the final concentration of 0.25 µg/mL for 1.5 h while shaking. Poly-HRP Streptavidin (Thermo Fisher Scientific, Loughborough, UK, part no. N200) was diluted 1:5000 in 1% BSA solution and incubated for 5 min while shaking. Color Reagent A and B containing 3,3′,5,5′-Tetramethylbenzidine (TMB) were mixed in a ratio of 1:1 (RnD Systems, Abingdon, UK, part no. DY007) and incubated on the plate for 2 min before being pipetted into a separate well to stop the reaction. Optical evaluation was performed using micro-volume spectrophotometer Genova Nano (Jenway, Staffordshire, UK) with 0.2 mm path length by measuring absorbance at 650 nm. The data were fitted with exponential function in Origin 9.1 software. Electrochemical measurements were performed by drop-casting the TMB solution on the PCB electrode surface and performing chronoamperometry at 0.1 DC bias vs. gold PCB quasi-reference electrode. The data point at 30 sec was used for quantification; three separate electrodes were used for obtaining the data. The change in blank was subtracted from the measurements before averaging. Hill fit was used to fit the data.

## 3. Results and Discussion

### 3.1. Removal of Surface Impurities in Commercially Manufactured PCB Electrodes

To use PCB electrodes as electrochemical sensors, the electrochemical behaviour of the bare electrodes must be repeatable and reproducible. Due to the low-cleanliness environment of the PCB manufacturing facilities, a level of impurities is present in the electrode surface after the production [5,9,10].

**Figure 1:**
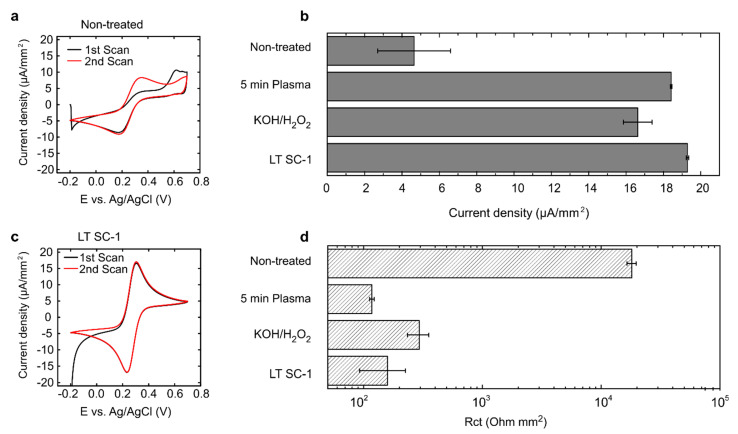
The effect of cleaning on electrode performance. (**a**) CVs in ferri-/ferrocyanide solution with non-treated PCB. (**b**) Comparison of current density obtained using CV in ferri-/ferrocyanide solution using multiple cleaning techniques by determining oxidation peak height. (**c**) CV in ferri-/ferrocyanide solution with PCB that underwent LT SC-1 cleaning treatment. (**d**) Comparison of Rct obtained using EIS in ferri-/ferrocyanide solution using multiple cleaning techniques. Bars represent the mean and error bars represent the SD, *N* = 4 in (**b**) and (**d**).

A pre-treatment step is therefore required to obtain a clean electroactive surface. Non-treated PCB electrodes revealed small currents in ferri-/ferrocyanide solution during the first scan and an increase in current density in the second scan to 4.6 µA/mm^2^, which remained stable in the following scans (Figure 1a,b). This indicates the presence of an insulating layer on the PCB electrodes, which is removed upon the potential increase, allowing redox reactions to occur.

Previous reports revealed that commercially fabricated PCB electrodes are covered with an organic layer [5,9]. There are multiple possibilities of organic layer removal, such as oxygen plasma treatment [11], which is an interesting possibility due to the availability of the technique in PCB manufacturing plants and the low environmental impact [12]. Five-minute treatment in oxygen plasma and subsequent CV analysis revealed that the current density increased to 18.4 µA/mm^2^ and the peak-to-peak separation decreased to 68 mV, indicating close-to-ideal behaviour for the ferri-/ferrocyanide couple. The average R_ct_ was 117 kΩmm^2^ (Figure 1b,d), while peak-to-peak separation in non-treated PCBs was 234 mV, which indicated that the insulating layer was not removed fully; this was also confirmed by EIS, where R_ct_ was found to be over 18 kΩmm^2^ (Figure 1d).

Another approach to removing organic contaminants on gold surfaces is a wet treatment with potassium hydroxide/hydrogen peroxide solution (50 mM KOH with 30% H_2_O_2_), as demonstrated previously [13]. CV and EIS analysis revealed 16.6 µA/mm^2^ oxidation currents and R_ct_, of 294 kΩmm^2^ while peak-to-peak separation remained similar at 71 mV. This indicates that the KOH/H_2_O_2_ treatment is an appropriate but not ideal process for the removal of impurities.

Another strategy for the removal of organic contaminants used in the semiconductor industry is standard clean 1 (SC-1), the first part of a multi-step RCA cleaning procedure, developed at the Radio Corporation of America in 1965 [14]. SC-1 includes immersion of the wafer in a mixture of hydrogen peroxide and ammonium hydroxide at 80 °C. This mixture initiates oxidative breakdown and dissolution of metallic ions such as copper, nickel, and chromium. Ammonium hydroxide acts as a complexing agent, holding Cu ions in solution, while hydrogen peroxide is an oxidising agent dissolving metallic copper [15]. Unfortunately, the PCB silkscreen and solder mask can be affected by the highly active SC-1 solution, so this process was adopted to clean PCBs by lowering the temperature of the SC-1 solution to RT and subsequent immersion of the PCB electrodes in acetone, isopropyl alcohol, and water to facilitate the complete removal of contaminants. This process will be referred to as low-temperature SC-1 clean (LT SC-1). Subsequent CV and EIS analysis in PCBs that underwent LT SC-1 clean revealed a consistent oxidation current density of 19.3 µA/mm^2^, average peak-to-peak separation of 69 mV, and an average R_ct_, of 158 Ωmm^2^ (Figure 1b–d). These data indicate that all the abovementioned procedures can be applied for PCB surface cleaning. It should, however, be noted that the performance of electrochemical sensors will strongly be affected by electrode reproducibility; hence, further methods and method combinations were explored to ensure highly reproducible electrode characteristics (Figures S2–S4). Among these, potential cycling in sulphuric acid, which is a widely used approach for the electrochemical cleaning of gold surfaces [16], was also explored as a post-processing step and demonstrated after increased capability to remove the organic layer in PCB electrodes (Figure S5).

**Figure 2:**
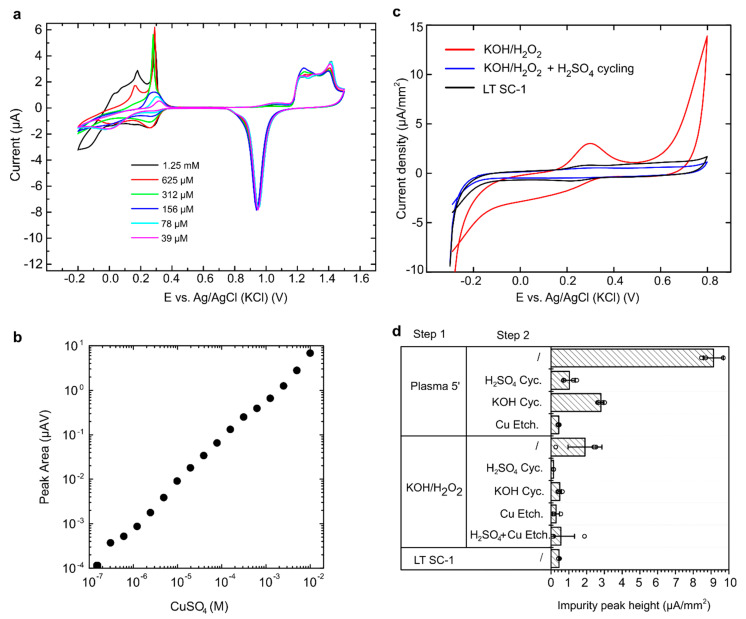
Determination of copper impurities in PCB electrodes. (**a**) CV curves obtained in sulphuric acid with increasing concentrations of CuSO_4_. (**b**) The relationship between CuSO_4_ concentration and peak area obtained in CV scans. (**c**) Analyses by CV in PBS, where Cu impurity peak is revealed at approximately 0.3 V. (**d**) The height of impurity peaks in PCBs that underwent various combinations of cleaning procedures (step 1 plus step 2). Bars represent the mean, error bars represent the SD, and empty circles represent individual data points.

Closer examinations of the CV scans in H_2_SO_4_ revealed inconsistent behaviour of the PCB electrodes (Figure S6). CV scans showed consistent oxidation and reduction of gold but an unknown peak appearing in the region between 0 and 0.4 V vs. Ag/AgCl (KCl). This could be the oxidation of copper impurities [17,18] or exposure of Cu under the Ni and Au. During the electroplating process, a certain level of impurities remains in the gold plating solution [15]. Although the maximum allowed level of Cu impurities in the electroplating bath is relatively low, Cu impurities are present on the electrode surface and can remain there in small quantities even after the electrode cleaning [5]. To confirm the source of the impurities, a PCB electrode that did not exhibit impurity peaks was exposed to increasing concentrations of CuSO_4_ in H_2_SO_4_ solution. Oxidation of Cu^2+^ ions was observed in the region of 0–0.3 V, presenting multiple peaks at high concentration (>300 µM); see Figure 2.

The observed peaks were integrated, and the peak area was plotted vs. the concentration of added CuSO_4_, revealing a linear relationship and confirming that the observed peaks were due to Cu impurities (Figure 2a,b).

To evaluate the level of Cu impurities, CVs of cleaned PCB electrodes were performed in PBS at high scan rates of 1 V/s. This revealed very high Cu peaks in PCBs cleaned with plasma (not shown) and lower Cu peak in PCBs cleaned with KOH/H_2_O_2_ treatment or LT SC-1 clean. Furthermore, when KOH/H_2_O_2_-treated PCBs were also cleaned with electrochemical cycling in H_2_SO_4_ solution, the Cu impurity peak disappeared completely (Figure 2c). A more systematic examination of different cleaning combinations was then tested in an effort to determine the most optimal procedure. Plasma treatment, KOH/H_2_O_2_ cleaning, and LT SC-1 clean were examined in combination with CV cycling in H_2_SO_4_, CV cycling in KOH, and commercially available copper etchant solution.

After plasma exposure, the average Cu peaks were very high at 9.1 µA/mm^2^ and dropped to 1.0 µA/mm^2^ with H_2_SO_4_ cycling, 2.8 µA/mm^2^ with KOH cycling, and 0.4 µA/mm^2^ with exposure to Cu etchant (Figure 2d). The data suggest that Cu impurities are best removed with a wet process; hence, plasma alone would not be suitable for PCB cleaning but can be combined with a Cu etch step to achieve appropriate surface characteristics.

KOH/H_2_O_2_ treatment revealed average Cu peaks of 1.9 µA/mm^2^, which dropped to below 1 µA/mm^2^ with every subsequent step (Figure 2d). LT SC-1 cleaning revealed low and very consistent levels of Cu impurities, with average Cu peaks of 0.4 µA/mm^2^. This confirms that a wet process is more suitable for the removal of Cu impurities.

**Figure 3:**
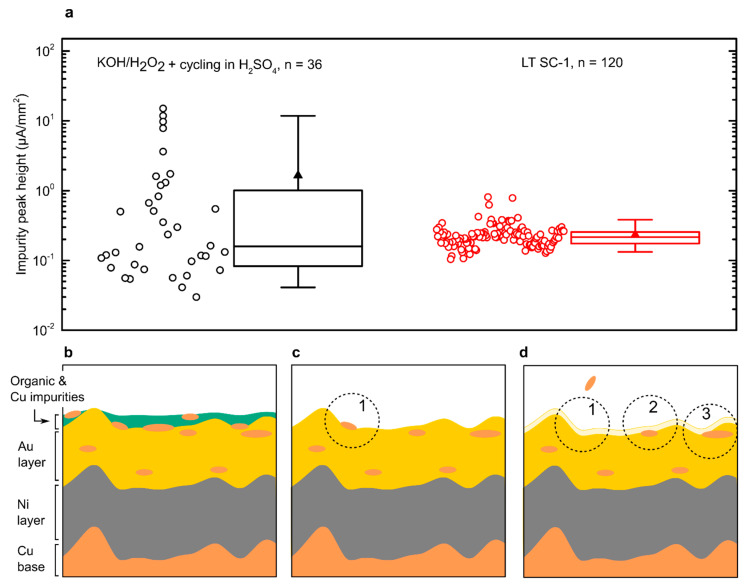
Electroactive impurity peaks in two different cleaning procedures and conceptual representation of PCB impurity removal process. (**a**) The height of impurity peaks in the larger sample number for two promising cleaning procedures. Empty circles represent individual electrodes, lines in a box represent the median, 25th and 75th percentile, the triangle is the mean value, whiskers represent the 5th and 95th percentile. (**b**) Non-treated PCB consisting of layers of a copper base, electroplated nickel, and gold. Cu impurities are found on top of the gold and within the gold layer. (**c**) PCB surface after wet KOH/H_2_O_2_ cleaning with removed organic and inorganic impurities. (**d**) Removal of the thin gold layer during CV cycling. Circle 1 represents the removal of Cu impurity and circles 2 and 3 represent newly formed exposure of the hidden Cu impurities within the plated electrodes.

To obtain greater insight into the reproducibility of the pre-treatment methodologies, assessment of a larger number of PCB electrodes was performed using two final candidates: LT SC-1 clean, due to its consistency, and KOH/H_2_O_2_ treatment followed by H_2_SO_4_ cycling, due to its excellent Cu removal capabilities. The latter procedure revealed almost complete removal of Cu peaks in some electrodes; however, other electrodes exhibited higher impurity peaks (Figure 3a). The range of impurities covered almost three orders of magnitude from 0.03 µA/mm^2^ to 15 µA/mm^2^ with an average of 1.6 µA/mm^2^. On the other hand, LT SC-1 cleaning revealed a lower average impurity peak of 0.2 µA/mm^2^ with the range from 0.1 µA/mm^2^ to 0.8 µA/mm^2^ using 120 PCB electrodes.

Additionally, Cu peak behaviour during H_2_SO_4_ cycling was not consistent. In some electrodes, Cu peaks decreased during CV cycling, while an increase in Cu peaks was observed with other PCBs. This indicated that two phenomena were occurring simultaneously.

We propose that this could be explained by copper contamination of the electroplated gold layer, depicted in Figure 3b–d.

Commercially fabricated PCBs are delivered with a layer of organic impurities, which prevent direct oxidation of ferri-/ferrocyanide couple or, more importantly, the formation of self-assembly monolayers (SAMs) and conjugation of capturing probes on the PCB surface (Figure 3b). Additionally, Cu is present in the initial layer [5], which can cause electroactivity of the PCB electrodes and can mask other desired processes occurring on the electrodes. A wet KOH/H_2_O_2_ treatment removed organic and the bulk of the inorganic impurities, with some remaining on the surface (Figure 3c), leading to Cu peaks observed in CV in PBS. Further CV cycling in sulphuric acid promotes the removal of Cu impurities, which can be seen as a cycle-dependent decrease in Cu peaks over time. However, during the scan, a thin layer of gold can be dissolved in the solution [19,20]. We postulate that this can expose Cu impurities in the interior of the gold layer, which is there as a consequence of the partly contaminated gold plating solution during the gold electrodeposition and was previously ‘hidden’ from the surface, protected from the wet cleaning process (Figure 3d). This is an increase in Cu peaks over time, observed by continuous scanning of PCBs in sulphuric acid. Besides the chemical surface composition, electrode roughness can possess a great effect on the electrochemical sensor’s performance [21,22,23].

### 3.2. Evaluation of PCB Surface Roughness

Extensive research can be found on strategies to increase the gold surface roughness to achieve high surface-to-volume ratios and enhance charge transfer properties [24]. However, high surface roughness can result in increased fouling properties [22,25,26]. Planar electrodes such as evaporated gold on glass or silicon substrates benefit from the smoothness of their respective substrates, leading to controllable smooth surfaces [27]. Although some applications utilise planar gold electrodes, a low-cost alternative solution predominantly includes screen-printed electrodes, which are known to possess very high surface roughness, due to the nature of their production [21,23]. PCB processing is expected to produce a surface of intermediate roughness, compared to these two commonly used technologies.

**Figure 4:**
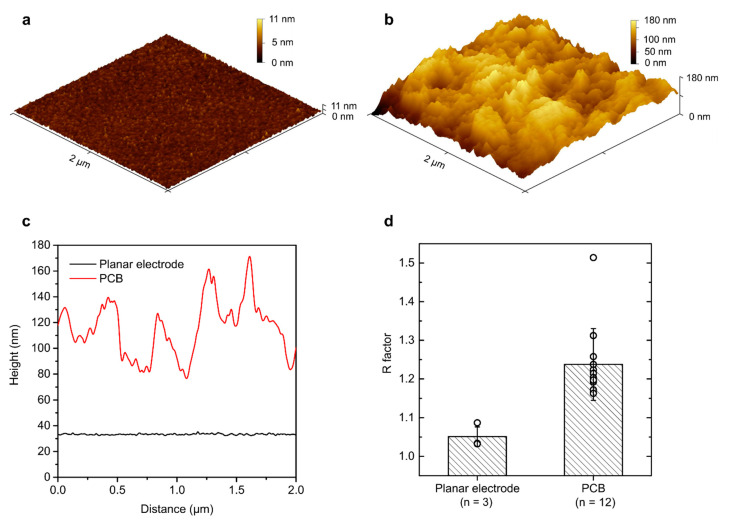
PCB surface roughness evaluation. (**a**) Example of the AFM profile for planar gold electrodes and (**b**) PCB electrodes. (**c**) The obtained profile from both representative samples. (**d**) Roughness factors obtained by CV scanning in H_2_SO_4_. Bars represent the mean, error bars represent the SD, and empty circles represent individual electrode datapoints.

To evaluate PCB electrodes, surface roughness was studied using atomic force microscopy as well as evaluated electrochemically. A thin-film, thermally evaporated planar gold electrode was compared to PCB electrodes. AFM revealed a smooth surface in planar electrodes, where all features fell within the 11 nm range. The average root mean square roughness from three measurements was 763.4 pm ± 62.8 pm. Five PCB electrodes were analysed with AFM, revealing the average root mean square roughness to be 20.99 nm ± 3.98 nm. This demonstrates that PCB electrodes are over 25-times rougher than planar gold electrodes. Representation of individual AFM scans can be seen in Figure 4a,b. Comparison of the extracted profiles revealed that PCB electrodes include features up to 100 nm in height (Figure 4c), compared to few-nm-level features in a planar gold electrode.

Surface characteristics were then also evaluated electrochemically. By CV cycling in sulphuric acid solution, the electrode’s electroactive surface area was evaluated and the roughness factor was calculated. The planar gold electrode revealed a mean roughness factor of 1.05, while the PCB electrode had an average roughness factor of 1.24 (Figure 4d). This is a small difference considering that the AFM results show 25-times higher surface roughness. Two factors contribute to the level of gold electroactivity: surface roughness and gold availability. Increased surface roughness increases the available area, but impurities covering the gold prevent direct contact with the solution and prevent gold oxidation, therefore decreasing the available surface area. Hence, electrochemical evaluations of PCB electrodes should be taken with caution, as small roughness factors do not necessarily indicate smooth surfaces but could be a consequence of impurities and decreased gold availability.

### 3.3. Electrochemical Protein Quantification Using Commercial PCB Electrodes

**Figure 5:**
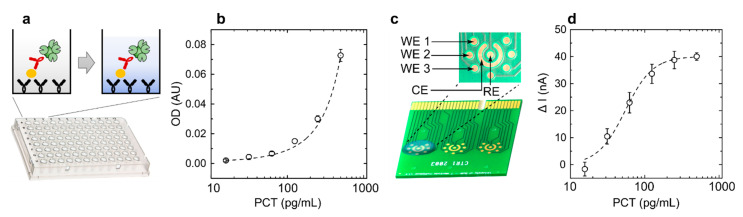
Optical and electrochemical quantification of PCT ELISA assay. (**a**) Conceptual representation of the ELISA assay performed in a 96-well plate, where the addition of TMB is seen as a colour change. (**b**) Optical detection of the colour change (*N* = 3). (**c**) Electrochemical set-up with gold PCB working electrodes (WE 1, 2, and 3) and shared counter (CE) and reference (RE) electrode. (**d**) Electrochemical detection of TMB using chronoamperometry (*N* = 3).

To demonstrate that commercial PCB electrodes can be used for the sensitive quantification of protein biomarkers, procalcitonin, a promising sepsis biomarker [28,29], was used as a model assay. PCT-based ELISA was constructed on a 96-well plate and TMB was measured optically and electrochemically (Figure 5).

Optical detection revealed an exponential increase in OD with increasing PCT concentrations, while electrochemical quantification revealed large changes in currents at low PCT concentrations, with an LoD of 31 pg/mL (or 2 pM). This demonstrated that the commercially manufactured PCB electrodes can be used for the sensitive electrochemical detection of catalysed TMB and consequently any protein when analysed with ELISA-based assays. This could be further expanded and integrated into microfluidic devices for low-cost quantification of any biomarker of interest, which can be quantified using ELISA-based systems.

Although the above system can be used for the quantification of an ELISA-based assay, many biosensors require immobilisation of the capturing probe on the electrode where the measurement is performed. An example of such a sensor is a label-free DNA sensor, presented in the next section.

### 3.4. Detection of DNA Using Commercial PCB Electrodes

**Figure 6:**
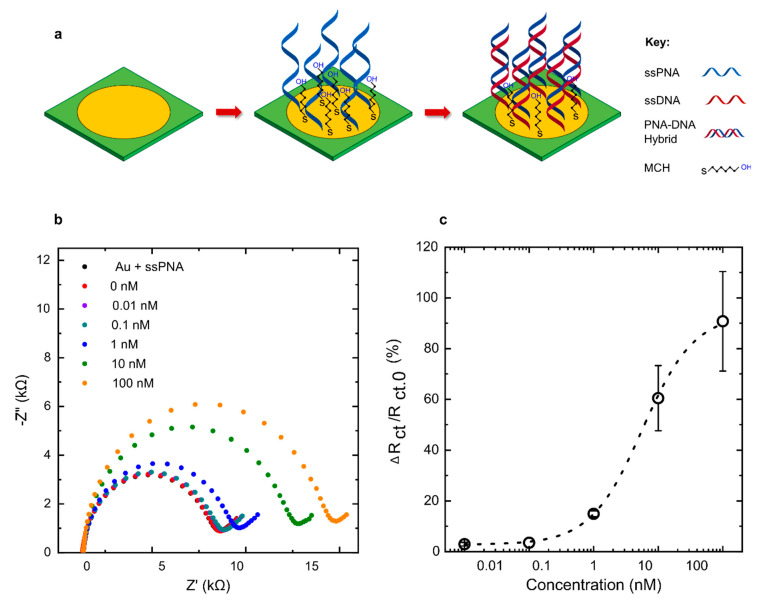
EIS-based PNA–DNA hybridisation assay on commercial PCB electrodes. (**a**) PCB gold electrode functionalised first with thiolated PNA probes and co-immobilised with MCH spacer molecules before direct hybridisation with ssDNA samples. (**b**) Typical Nyquist plots obtained for PNA–DNA hybridisation using thiol surface chemistry on PCB with five concentrations of target ssDNA and blank sample. (**c**) A calibration curve for the percentage change in R_ct_ using five concentrations of ssDNA in buffer (*N* = 3).

To evaluate the feasibility of capture probe immobilisation on commercially fabricated PCB electrodes, a biosensor was fabricated using single-stranded peptide nucleic acids (ssPNA) as biological probes for the detection of genomic *E. coli* ssDNA. The sequences came from genes that code for an *E. coli*-specific virulent factor called fimbriae protein, which aids in the invasion of the host system and is highly conserved within the O157:H7 serotype genome [30]. Thiolated PNA probes were immobilised on pre-cleaned commercial PCB electrodes and the electrochemical impedance spectra were recorded upon measurement of a blank as well as five concentrations of ssDNA (Figure 6). The electrochemical impedance spectra data show a significant change between 0.01 and 100 nM. The non-linear curve fit shows a significant R-squared value of 0.95. The limit of detection (LoD) was calculated to be 110 pM using an optimised co-immobilisation molar ratio of 1:15. The Nyquist plot shows a dynamic range between 100 pM and 10 nM, with an observed saturation effect between 10 and 100 nM.

We believe that the higher error at high concentrations of the DNA target can be attributed to variation in the surface roughness and immobilised probe density. This seems to be less prominent at a lower concentration of target DNA, as a highly reproducible change in Rct (approximately 20%) can be seen at 1 nM DNA concentration. Due to the surface morphology leading to variability in SAM formation, the exact probe density varied from electrode to electrode. As higher concentrations of target DNA were introduced to the sample, the sensor surface began to saturate, and the probe density can have a major effect on the obtained Rct. This can be seen as a larger variation in the sensor response at a high target concentration.

## 4. Conclusions

The use of commercially employed processes for PCB manufacturing in constructing sensitive and reliable electrochemical sensors could enable low-cost diagnostic microsystems, which could be scaled and produced locally, using existing manufacturing infrastructure. Nevertheless, this required reliability of electrochemical biosensors is underpinned by the reproducible physicochemical characteristics of the sensing electrodes and the respective processes used for their construction. Gold bioavailability and the lack of contaminants in electrode surfaces are crucial in the final device performance as they directly affect the surface chemistry and the final readout of the sensor. Detailed electrochemical characterisation revealed suitable electrode cleaning procedures, which provide reproducible electrode characteristics and effective removal of electroactive contaminants.

Gold-plated PCB electrodes have a considerably higher surface roughness (RMS roughness of ca. 20 nm) compared to cleanroom-fabricated thin-film gold electrodes (RMS roughness of ca. 1 nm) but, at the same time, orders of magnitude smaller than the widely used screen-printed ones (RMS roughness of 1 µm) [24]. As surface roughness is known to have a profound effect on biosensor performance, this indicates that sensors that cannot be implemented in commercially screen-printed electrodes due to their high surface roughness can be implemented on PCB electrodes, offering a low-cost alternative to the high-specification evaporated ones.

Finally, reliable electrochemical detection using such optimised, commercial PCB sensing electrodes was demonstrated. Label-free detection of DNA based on PNA probes is shown, along with quantification of the protein biomarker PCT, achieving clinically relevant limits of detection. With this knowledge, electrochemical sensors can now be constructed on commercially manufactured PCB electrodes for a multitude of biomarker quantification applications, paving the way for reliable commercial Lab-on-PCB biomedical diagnostic platforms.

## Figures and Tables

**Table 1:** Cleaning procedures used in the study.

Cleaning Method	Procedure
Oxygen plasma treatment	3, 5, or 10 min at 100 W and 0.2 mbar (Diener Zepto System, Diener electronic, Ebhausen, Germany).
KOH/H_2_O_2_ treatment	Immersion in a solution of 30% H_2_O_2_ and 50 mM KOH for 10 min.
LT SC-1 clean	Step 1: Immersion in a solution of 30% NH_4_OH, 30% H_2_O_2_, and MQ water in a ratio of 1:1:5 for 15 min. Step 2: Immersion in >99% acetone solution for 5 min. Step 3: Immersion in >99% IPA solution for 5 min. Step 4: Immersion in MQ water for 5 min.

## Data Availability

Data available from authors upon request.

## Supplementary Materials

The following are available online at https://www.mdpi.com/article/10.3390/mi12070793/s1, Table S1, Additional cleaning steps used in combination with previously described cleaning procedures. Figure S1, PCB boards used to characterise PCB electrodes. The PCB with eight electrodes (WEs) which connect to a PCI express slot-type connector. Figure S2, Current obtained from CV in ferri-/ferrocyanide with a combination of multiple cleaning steps. Bars represent the average and error bars represent the SD, *N* = 4. Figure S3, Peak-to-peak separation value obtained from CV in ferri-/ferrocyanide with a combination of multiple cleaning steps. Bars represent the average and error bars represent the SD, *N* = 4. Figure S4, Charge transfer resistance values obtained from EIS in ferri-/ferrocyanide solution with a combination of multiple cleaning steps. Bars represent the average and error bars represent the SD, *N* = 4. Figure S5, Charge transfer resistance obtained with PCB electrodes in ferri-/ferrocyanide solution with different treatments. Bars represent the mean and error bars represent the SD, *N* = 4. Figure S6, Examples of CV curves obtained by electrochemical polishing of PCB electrodes in sulphuric acid. Ox represents the peak demonstrating formation of gold oxide layer and Red represents the reduction of gold oxide in a reverse CV scan.
