# Supplementary material for: Utilising Commercially Fabricated Printed Circuit Boards as an Electrochemical Biosensing Platform
Source: Micromachines (Basel). 2021 Jul 3;12(7):793. doi: 10.3390/mi12070793 (PMC8305449; doi:10.3390/mi12070793)
Supplement: Supplementary file 1 [file micromachines-12-00793-s001.zip › micromachines-1265218-supplementary.pdf]

# Supporting information

# Utilizing Commercially Fabricated Printed Circuit Boards as an Electrochemical Biosensing Platform

*Uroš Zupančič, Joshua Rainbow, Pedro Estrela, and Despina Moschou**

Centre for Biosensors, Bioelectronics and Biodevices (C3Bio) and Department of Electronic & Electrical Engineering, University of Bath, Claverton Down, Bath, Somerset, BA2 7AY, UK

Uroš Zupančič (email: uz206@bath.ac.uk)
Joshua Rainbow (email: jr993@bath.ac.uk)
Pedro Estrela (email: p.estrela@bath.ac.uk)
*Despina Moschou (email: dm855@bath.ac.uk, phone: +44 (0) 1225 383245

PCBs used in the cleaning study are shown in **Figure S1**. In the effort to determine the optimised version of cleaning steps required for removal of insulating organic layer from the PCB surface, multiple combinations were examined and evaluated in terms of obtained current (**Figure S2**), peak-to-peak separation values (**Figure S3Error! Reference source not found.**) and charge transfer resistance (**Figure S4**). Other cleaning methods and combinations are described in **Table S1**.

**Table S1**. Additional cleaning steps used in combination with previously described cleaning procedures.

| Cleaning method | Procedure |
|---|---|
| Hydrogen peroxide & 500 mM KOH | Immersion in a solution 30% $H_2O_2$ & 500 mM KOH for 10 min |
| KOH cycling | 15 CV cycles between -1.1 and 0.85 V vs. Ag/AgCl (KCl) at 100mV/s in 50 mM KOH . |
| $H_2SO_4$ cycling | 30 CV cycles between -0.2 and +1.5 V vs. Ag/AgCl (KCl) at 200 mV/s in 50 mM $H_2SO_4$. |
| KOH sweep | Single potential sweep in 50mM KOH from -0.2 to -1.2 V vs. Ag/AgCl (KCl) at 50 mV/s. |

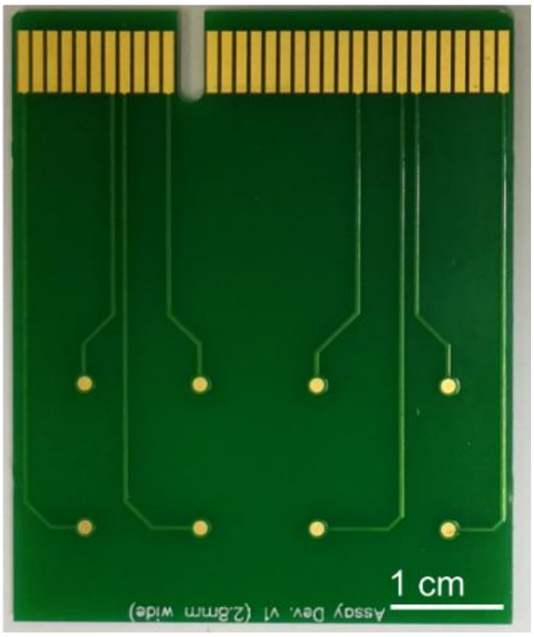


**Figure S1**. PCB boards used to characterise PCB electrodes. The PCB with eight electrodes (WEs) which connect to a PCI express slot-type connector.

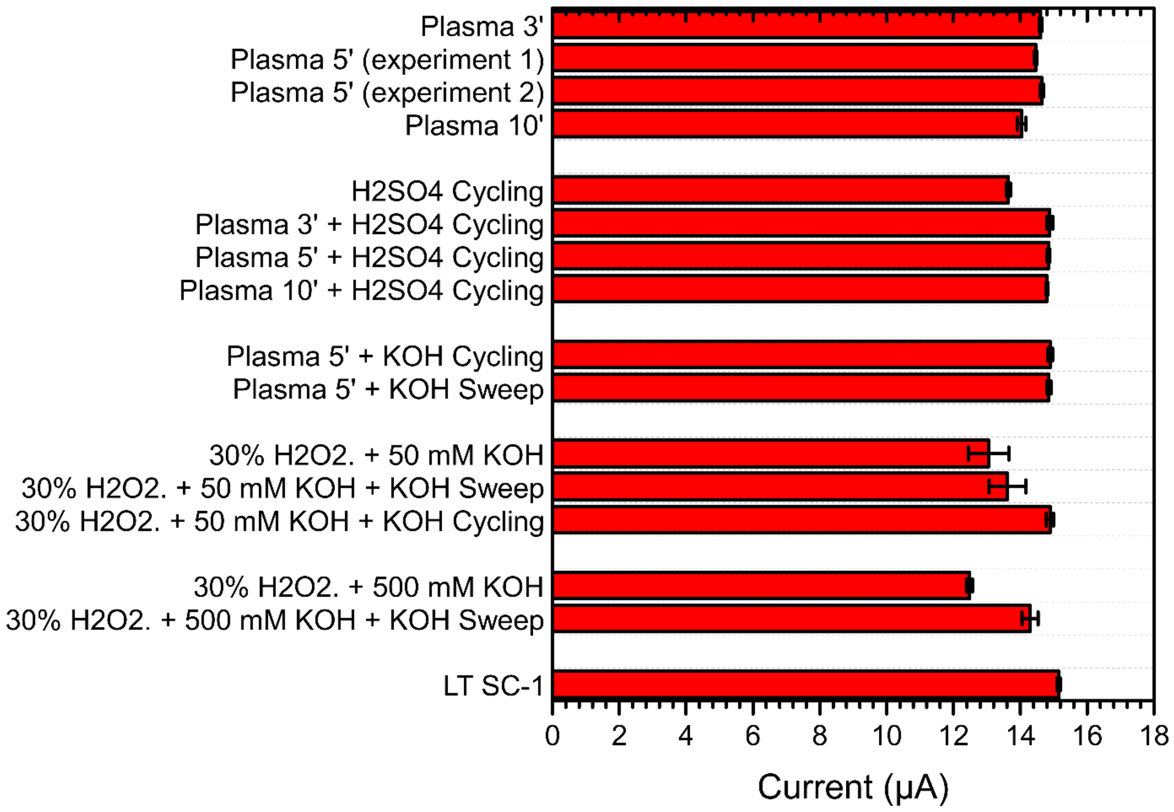


**Figure S2.** Current obtained from CV in ferri-/ferrocyanide with a combination of multiple cleaning steps. Bars represent the average and error bars represent the SD, N = 4.

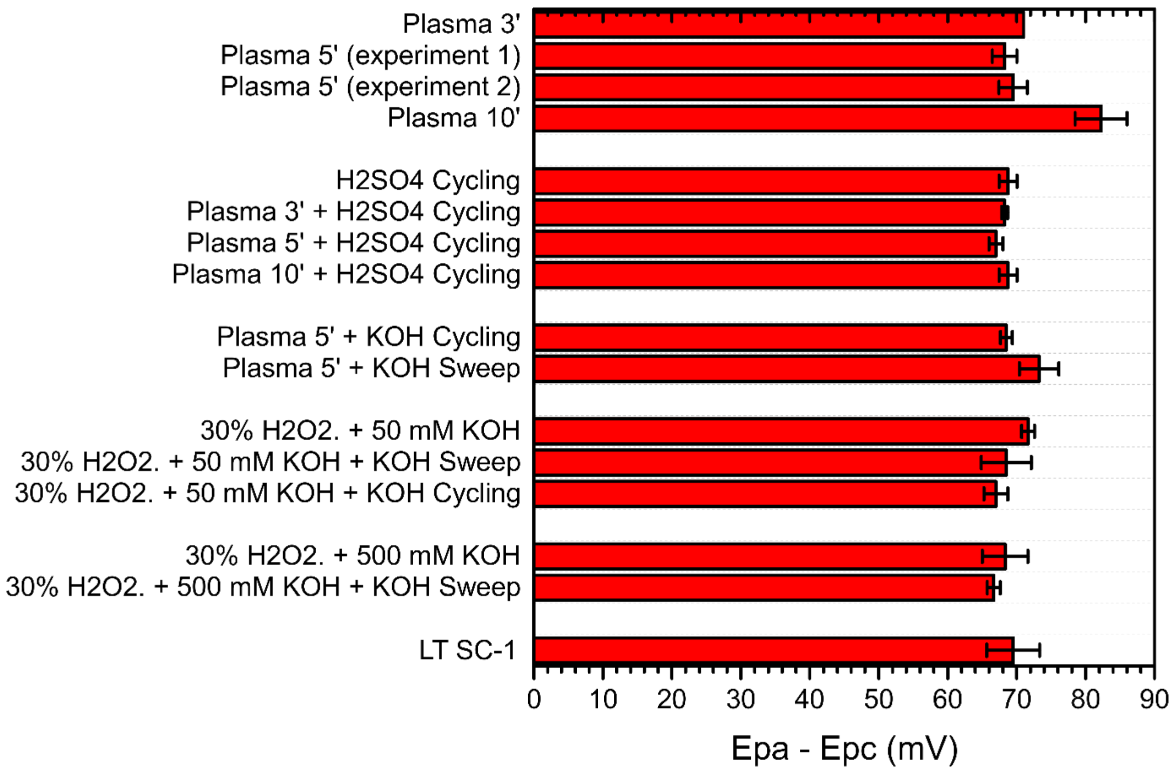


**Figure S3.** Peak-to-peak separation value obtained from CV in ferri-/ferrocyanide with a combination of multiple cleaning steps. Bars represent the average and error bars represent the SD, N = 4.

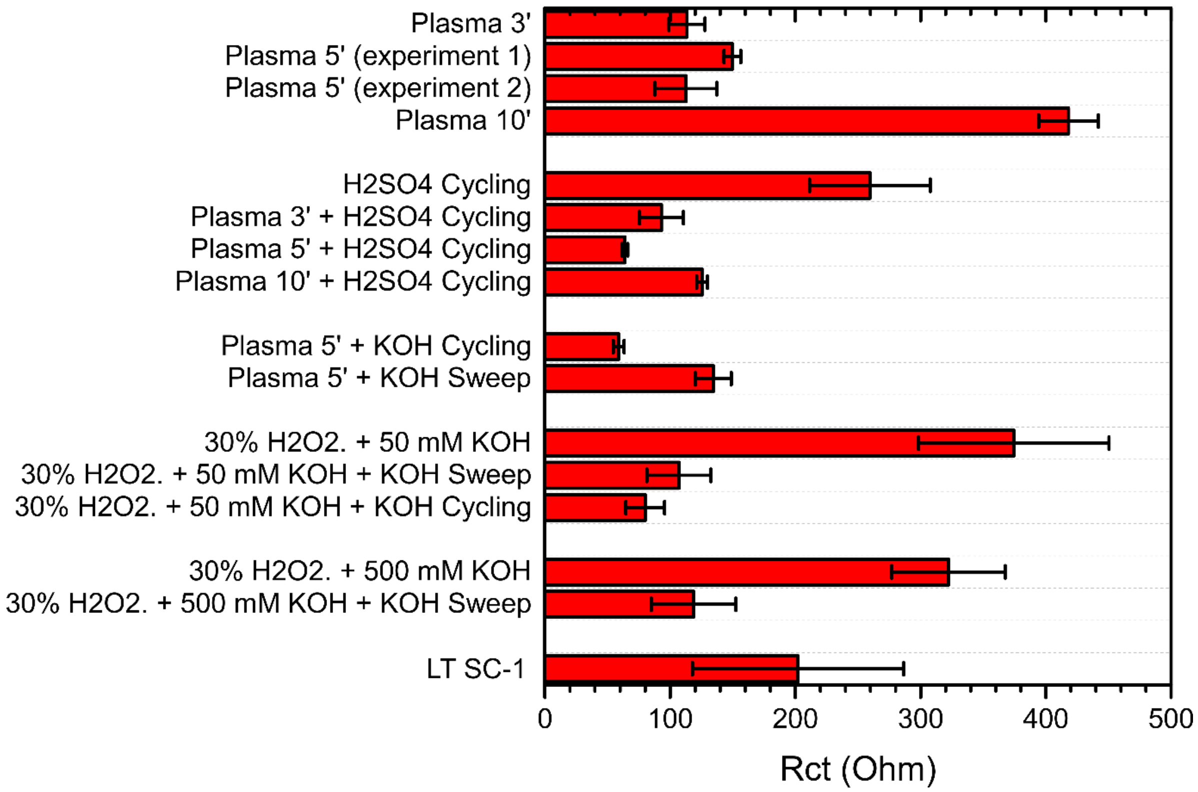


**Figure S4**. Charge transfer resistance values obtained from EIS in ferri-/ferrocyanide solution with a combination of multiple cleaning steps. Bars represent the average and error bars represent the SD, N = 4.

Charge transfer resistance from EIS scan in ferri-/ferrocyanide solution was used to evaluate different oxygen plasma exposure times of 3 min, 5 min and 10 min (**Figure S5**). Surprisingly, longer exposure times increased the $R_{ct}$, which could be an effect of plasma attacking the PCB solder mask and cross contaminating the exposed gold electrodes, as a slight change in solder mask colour was observed after 10 min treatment with oxygen plasma. Sulfuric acid cycling did not reveal large differences in the obtained $R_{ct}$ values when compared to 3 min plasma treatment but caused the decrease in the average $R_{ct}$ values drop from 117 $\Omega mm^2$ to 50 $\Omega mm^2$ in case of 5 min plasma treatment. This effect was pronounced at 10 min plasma treatment times demonstrating the sulfuric acid cycling facilitates removal of impurities. Similar $R_{ct}$ vales were obtained using the KOH cycling, demonstrating both options are suitable.

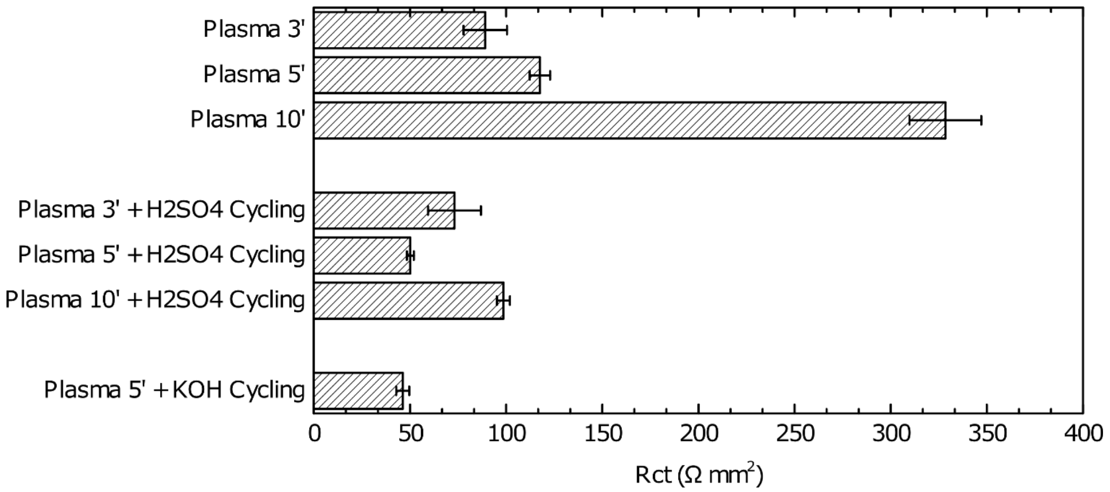


**Figure S5.** Charge transfer resistance obtained with PCB electrodes in ferri-/ferrocyanide solution with different treatments. Bars represent the mean and error bars represent the SD, N = 4.

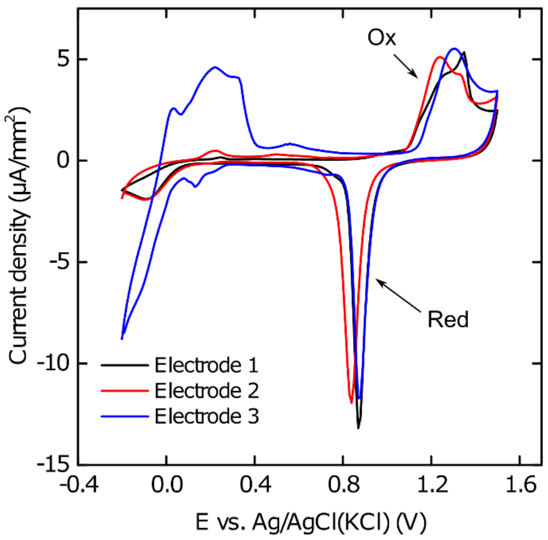


**Figure S6.** Examples of CV curves obtained by electrochemical polishing of PCB electrodes in sulfuric acid. Ox represents the peak demonstrating formation of gold oxide layer and Red represents the reduction of gold oxide in a reverse CV scan.
